# Patient-Reported Outcomes as a Tool for Involvement in Metastatic Melanoma Care

**DOI:** 10.2340/1651-226X.2025.43465

**Published:** 2025-08-12

**Authors:** Pernille Christiansen Skovlund, Ditte Minet Karkov, Charlotte Gjørup Pedersen, Annesofie Lunde Jensen

**Affiliations:** a Department of Oncology, Aarhus University Hospital, Aarhus, Denmark; b Department of Public Health, Aarhus University, Aarhus, Denmark; c Steno Diabetes Center Aarhus, Aarhus University Hospital, Aarhus, Denmark; d Department of Clinical Medicine, Aarhus University, Aarhus, Denmark

**Keywords:** Patient participation, patient reported outcome measures, melanoma, physician-patient relations, health communication

## Abstract

**Background and purpose:**

Patients with metastatic melanoma live longer than a decade ago and have limited contact with the healthcare system. This requires a focus on their ability to manage their health. Patient involvement can contribute to this. The use of Patient-Reported Outcome (PRO) may facilitate patient involvement in clinical encounters, especially in the form of enhanced communication between patient and clinician. The purpose of the study was to investigate the association between active use of PRO and patient involvement for patients with metastatic melanoma.

**Patient/material and methods:**

This study was based on data from a non-randomized controlled study, in which Danish patients with metastatic melanoma were assigned to either an intervention (PRO actively used as a dialog tool in consultations throughout a year) or control group (standard treatment), based on geographic affiliation. The outcome in the present study was patient involvement, measured with five indicators of patient involvement. Linear regression models were used to estimate the crude and adjusted association between intervention and patient involvement at 3, 6, and 12 months.

**Results:**

A total of 237 patients were included, 114 patients in the intervention group and 123 patients in the control group. Adjusted mean difference between intervention and control group was 1.54 (0.24; 2.83) at 6 months and 1.32 (0.06; 2.59) at 12 months (*p* < 0.05). Improvement was observed in just one indicator of patient involvement, specifically ‘dialog between patient and physician’.

**Interpretation:**

Using PRO actively as a dialog tool in consultations can contribute to improved patient involvement for patients with metastatic melanoma.

## Introduction

Melanoma accounts for approximately 1% of all skin cancers and significantly contributes to skin cancer-related mortality due to its rapid growth and high potential for metastasis [1]. The incidence of melanoma is rising in several predominantly fair-skinned countries [1]. Treatment of melanoma typically begins with surgery, while individuals with metastasis are referred to oncological treatment including radiation therapy, immunotherapy, targeted therapy, and/or chemotherapy [2]. A significant advancement in treatment has increased the 5-year survival rates for patients with metastatic melanoma [3]. Unfortunately, better treatment comes with a higher degree of toxicity [3]. In addition, individuals with melanoma may experience limitations in their lifestyle and activities, as well as anxiety and thoughts about their prognosis [4]. Oncological treatments are often administered in outpatient settings, which means limited contact with health professionals. Reduced communication with health professionals in outpatient settings and improved survival rates require patients to take on the responsibility of self-managing their health and addressing treatment-related side effects linked to metastatic melanoma [5]. This highlights the importance of self-management, a core element of patient involvement [6].

Patient involvement is a complex and dynamic concept without a specific definition. It is considered a partnership approach like patient-engagement, patient-participation, and patient-centeredness [7]. When a patient and a physician actively engage in a dialog based on trust, respect, and shared decision-making, patient involvement is enhanced [8]. Patient involvement is expected to benefit from better clinical outcomes for the patient [8]. Research has shown that patients with metastatic cancer prefer to be involved in decision-making about their health [9, 10]. This could be achieved by actively using Patient-Reported Outcomes (PRO) to facilitate patient involvement and collaboration between patients and clinicians [11, 12]. PRO is a questionnaire that directs reports from patients on their health status, covering physical and emotional symptoms as well as health-related quality of life [13]. Most studies primarily focus on patient–clinician communication as an outcome measure for patient involvement [14, 15]. Only a few studies have investigated patient involvement in a broader sense [16–18]. Among them, one study specifically found that using PRO in consultations expanded dialog and prompted patients to ask questions, and share their experiences and concerns. This led to high levels of reported patient involvement [16].

PRO can serve as a measurement tool for research, quality improvement, and clinical practice [13]. Patients with cancer have shown a strong willingness to complete PRO questionnaires, perceiving them as relevant to their condition and integral to their healthcare [19]. Both patients and physicians value PRO as essential tools in healthcare [19, 20]. When used actively as a dialog tool in consultations, PRO can create a shared focus on problems, preferences and needs [19, 20]. It may also support and enhance patient–physician communication, by prompting discussion and streamlining consultations [11, 14, 15]. The association between PRO and patient involvement during consultations for metastatic melanoma is unknown; therefore, it is crucial to determine whether patients perceive their active engagement in consultations to be better when using PRO actively compared to when not using PRO. Thus, this study aimed to investigate the association between patient involvement and the use of PRO as a dialog-based tool in patient–physician communication for patients with metastatic melanoma.

## Patients/material and methods

The CONSORT 2010 checklist has been followed for reporting of this study and can be found in the Supplementary material.

### Context of study

This study is based on data collected in a controlled clinical intervention trial (hereinafter referred to as the controlled study) [5]. The primary outcome in the controlled study was patient activation, as measured using the questionnaire Patient Activation Measurement (PAM). Secondary outcomes were health-related quality of life, self-efficacy, and patient-perceived efficacy in the patient–physician interaction. Furthermore, data on patient involvement were collected as a secondary outcome, but these results have not yet been analyzed or reported. In the present study, the focus is only on patient involvement. The PRO questionnaires were selected in collaboration with patient representatives during the intervention development. Patients completed PRO questionnaires at baseline and 3-, 6-, and 12-months following the intervention or after recruitment in the control group [5].

### Included patients in the controlled study

Patients were recruited during consultations at an outpatient oncology clinic at three Danish hospitals (Aarhus University Hospital, Odense University Hospital, and Herlev Hospital) from June 2017 until July 2019. Patients were included if they were diagnosed with metastatic melanoma, referred to oncological treatment (irrespective of referral time or disease severity), their life expectancy exceeded 2 months, were Danish-speaking, and consented to complete repeated questionnaires. A total of 279 patients were included at baseline in the controlled study [5]. However, not all 279 patients answered questions about patient involvement at least once during study participation.

### Description of the intervention

The intervention involved patients from Aarhus University Hospital, with participants from Odense and Herlev hospitals serving as control groups [5]. The intervention involved the active use of PRO as a dialog-based tool in patient–physician consultations. Physicians received a 1-h training session, ad hoc training, and a manual on using PRO to promote patient-centered communication in consultations [21]. Patients completed the PRO questionnaire prior to each consultation, either at home or using a tablet in the waiting room. The PRO included the European Organization for Research and Treatment of Cancer-Core Quality of Life Questionnaire, version 3.0 (EORTC QLQ-C30), the Hospital Anxiety and Depression Scale (HADS), and open-ended questions about concerns and the three most important issues to discuss in consultation [5]. All questionnaire responses were recorded in real-time into the patient’s electronic medical records using the Ambuflex (WestChronic system) – a web-based dialog and decision support tool consisting of digital questionnaires completed by patients [13]. Data were visually presented in the electronic medical records, with colors indicating the severity of each response; green denoted no problem, yellow represented mild, orange signified moderate, and red indicated a significant burden for the patient [5]. This provided physicians with an overview of the patient’s symptoms, functioning, and concerns, and served as the starting point of the consultation.

### Description of the control group

Patients in the control group received usual care, which included a patient-centered approach but did not incorporate PRO as a dialog tool or other systematic methods for patient involvement in consultations.

### Outcome in current study

The outcome of this current study was patient involvement, assessed with a five-item valid questionnaire. The five PRO-questions for patient involvement have been developed and validated in Danish [22, 23]. Through the five PRO-questions participants were asked about their level of agreement with the following five statements: (1) ‘The healthcare professionals asked questions about my experiences with the disease’, (2) ‘I talked to healthcare professionals about the questions and concerns that I had’, (3) ‘The healthcare professionals invited me to ask questions and talk about my concerns’, (4) ‘I was consulted when decisions about my plans were made’, and (5) ‘I talked adequately to healthcare professionals about how I manage my condition’. Each item was rated on a five-point scale from 0 (do not know) to 5 (to a very high degree). The sum score for each item spans from 0 (worst) to 25 (best) [22, 24]. Questionnaires completed at 3, 6, or 12 months after recruitment were included. Baseline data were not used because the questionnaire referred to the previous consultation, and not all patients had one before entering the study.

### Covariates

Covariates and potential confounders were based on the literature and obtained from the questionnaires. Sex, age, education level, cohabitation status, employment status, and time from diagnosis with metastatic melanoma to enrolment in the controlled study were included [5]. Those factors have been associated with patient involvement [22, 25].

### Statistical method

Data on sociodemographic and treatment reported at the study’s entry were presented as proportions and percentages for categorical variables and as means for continuous variables, for both the intervention and control groups.

The individual items were examined separately, examining the distribution of responses per option. Differences between the intervention and control groups were tested using the chi-square test and presented with p-values. A sum score for patient involvement was estimated if three out of the five statements were answered at 3, 6, or 12 months for both the intervention and the control groups. In case of two or fewer missing items, the missing value was replaced with the mean score of the answered items at the time of response [26]. At 3, 6, and 12 months, separate linear models were fitted with and without adjustment for the potential confounders [27, 28]. Patients with missing values on covariates were excluded from the multiple linear analyses due to few and non-systematic missing values. Statistical software Stata version 17.0 (StataCorp, Texas, USA) was used for all statistical analysis [29].

## Results

**Figure 1:**
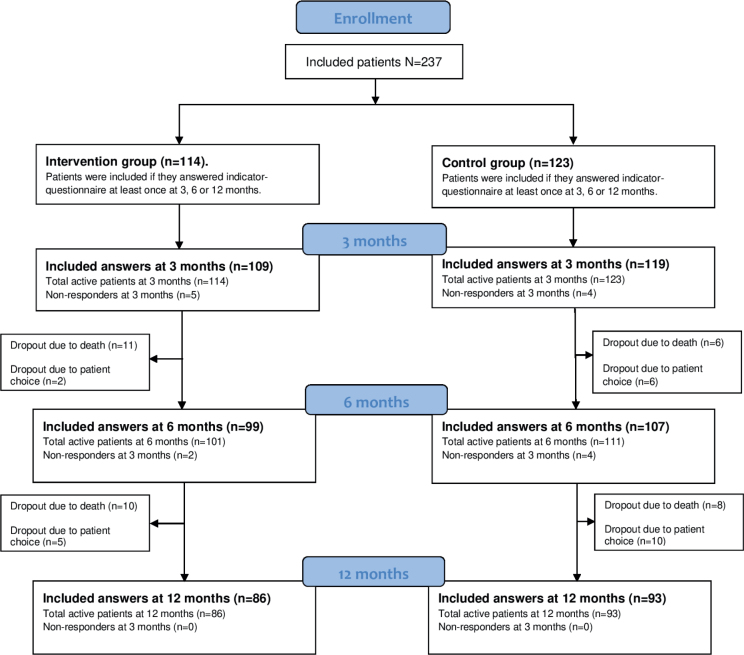
Participant flow.

Out of 279 patients in the controlled study, a total of 237 patients (85%) entered the present study (Figure 1). At 3 months, the response rate was 96.2% (*n* = 228) out of the 237 patients (81.7% of all patients in the controlled study answered the questions about patient involvement at 3 months, of which 47.8% (*n* = 109) were included in the intervention group and 52.2% (*n* = 119) in the control group). At 6 months, the response rate was 86.9% (*n* = 206) (73.8% of all patients in the controlled study) of which 48.0% (*n* = 99) were included in the intervention and 51.9% in the control group. At 12 months, the response rate was 75.5% (*n* = 179) (64% of all patients in the controlled study), with 48.0% (*n* = 86) in the intervention group and 52.0% (*n* = 93) in the control group (Figure 1).

### Loss to follow-up

Overall, the reasons for patients’ loss to follow-up were death (*n* = 35, with *n* = 21 in the intervention group and *n* = 14 in the control group) and dropouts (*n* = 23, with *n* = 7 in the intervention group and *n* = 16 in the control group) (Figure 1). From 3 months to 12-month follow-up, 6% (*n* = 7) in the intervention group and 13% (*n* = 16) in the control group dropped out. Statistical analysis for dropouts was not performed due to the low number of dropouts.

### Participant characteristics

Table 1 shows the characteristics of participants in the intervention and control groups. Based on percentages, it was observed that participants in the intervention were more often males (64.0% males in intervention. 44.7% males in the control group) and had < 6 months from diagnosis to study inclusion than participants in the control group (48.3% < 6 months in intervention. 20.3% < 6 months in control group).

**Table 1:** Participants’ characteristics in the intervention group and control group at the time of study entry.

Participants characteristics	Intervention *N* = 114 *n* (%)	Control *N* = 123 *n* (%)
Age	62.8 (12.1)^a^	64.7 (12.1)^a^
Gender		
Female	41 (36.0)	68 (55.3)
Male	73 (64.0)	55 (44.7)
Education level*		
None, short courses	19 (16.7)	22 (17.9)
Skilled worker	51 (44.7)	44 (35.8)
Higher education (≤ 4 years)	27 (23.7)	39 (31.7)
Higher education (> 4 years)	15 (13.2)	16 (13.0)
Missing	2 (1.8)	2 (1.6)
Employment status		
Employed	52 (45.6)	51 (41.5)
Unemployed (incl. pensioner)	62 (54.4)	72 (58.5)
Cohabitation		
Single	25 (22.1)	31 (25.2)
Partner	88 (77.9)	92 (74.8)
Comorbidity		
≤ 1	90 (79.0)	101 (82.8)
≥ 2	24 (21.1)	21 (17.2)
Time from diagnosed metastasis to baseline*		
< 6 months	55 (48.3)	25 (20.3)
≥ 6 months	58 (50.9)	95 (77.2)
Missing	1 (0.9)	3 (2.4)
Treatment		
Immunotherapy	85 (74.6)	89 (73.6)
Other**	23 (20.2)	23 (19.0)
Do not know	6 (5.3)	9 (7.4)

Note. ^a^Mean (SD),

* Variable with missing values. Number of missing values for intervention and control group combined < 5%.

** Targeted therapy, clinical trials (five patients (4%) in the control group, none in the intervention group), or best supportive care.

**Figure 2:**
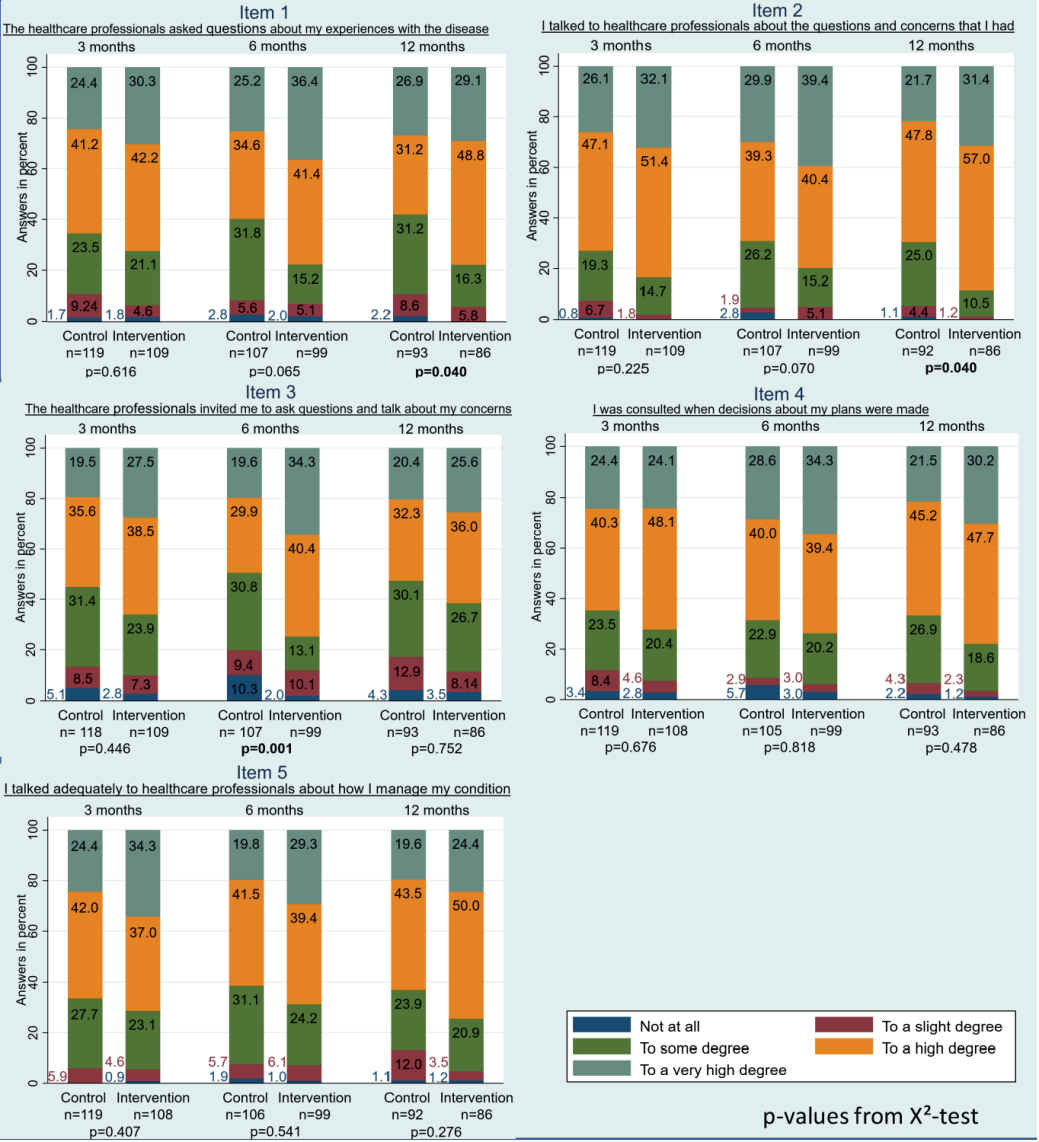
*P*-values from X^2^-test.

At 3 months, a minor difference in crude mean patient involvement was observed between the intervention and control groups. However, this was not statistically significant in the adjusted analysis (95% CI −0.10 to 2.13; *p* = 0.075) (Table 2). At 6 months, participants in the intervention group had statistically significantly higher mean scores (adjusted) for patient involvement compared to participants in the control group (95% CI 0.24 to 2.83; *p* = 0.021). The same result was seen at 12 months (95% CI 0.06 to 2.59; *p* = 0.041) (Table 2). Regarding the individual items, Figure 2 shows that an effect was observed for items 1 and 2 after 12 months (*p* < 0.05), while item 3 showed an effect after 6 months (*p* < 0.05), but this effect was no longer measurable at 12 months (*p* > 0.05). These three items addressed (1) ‘The healthcare professionals asked questions about my experiences with the disease’, (2) ‘I talked to healthcare professionals about the questions and concerns I had’, and (3) ‘The healthcare professionals invited me to ask questions and talk about my concerns’. No effect was observed between the intervention and control groups for the other items.

**Table 2:** Crude and adjusted mean differences in patient involvement for the intervention and control group at 3-, 6-, and 12 months.

Time	Intervention			Control			Crude		Adjusted*				*p*
	*n*	Mean	95% CI	*n*	Mean	95% CI	Mean	95% CI	n** intervention	n** control	Mean	95% CI
3 months	109	19.72	(18.99; 20.45)	119	18.84	(18.14; 19.54)	0.88	(-0.13; 1.89)	108	114	1.01	(-0.10; 2.13)	0.075
6 months	99	20.03	(19.21; 20.85)	107	18.59	(17.81; 19.39)	1.43	(0.29; 2.57)	97	102	1.54	(0.24; 2.83)	**0.021**
12 months	86	19.88	(19.10; 20.67)	93	18.54	(17.80; 19.30)	1.34	(0.25; 2.42)	86	89	1.32	(0.06; 2.59)	**0.041**

* Adjusted for age, gender, education level, employment status, cohabitation, comorbidities, and time from diagnosed with metastasis to enrollment.

** Differences in *n* for crude and adjusted estimates due to complete case analysis as a strategy for handling missing values.

Statistically significant values (p<0,05) are writen in bold.

## Discussion and conclusion

This study investigated the association between the active use of PRO as a dialog tool during consultations and patient involvement in patients with metastatic melanoma 12 months after the consultation. Our findings showed that patients who received the intervention had significantly higher involvement scores than those in the control group at 6 and 12 months, with no difference observed at 3 months. Notably, three items stood out, as the intervention group reported higher agreement with the statements: ‘The healthcare professionals asked questions about my experiences with the disease’, ‘I talked to healthcare professionals about the questions and concerns I had’, and ‘The healthcare professionals invited me to ask questions and talk about my concerns’.

From a patient perspective, the results of the intervention indicate its potential to enhance patient involvement. This result aligns with findings from other studies that involve the perspectives of both physicians and patients [14, 30, 31]. Moreover, it is clinically relevant to consider the average level of patient involvement, which ranges from 0 to 25 in total. In our study, no participants reported a complete lack or very low level of involvement, regardless of whether they were in the intervention or control group. It should also be considered whether a difference of less than two points in the total score meaningfully impacts the level of involvement for the individual patient. This question has not been further examined in this study. Furthermore, considerations about the implementation of PRO as a dialog tool also need to be taken into account. Cost–benefit analyses regarding resources (e.g. time, money, manpower) that go into the clinical use of PRO and the respondent burden are highly warranted.

It is interesting to note that the intervention showed a long-term effect on only two of the five items: (1) ‘The healthcare professionals asked questions about my experiences with the disease’, and (2) ‘I talked to healthcare professionals about the questions and concerns I had’. Meanwhile, item (3) ‘The healthcare professionals invited me to ask questions and talk about my concerns’, was prominent at 6 months but showed no long-term effect. This indicates that both physicians in the intervention and control groups have room for improvement, particularly when dealing with patients who would prefer an invitation to ask questions. Patients who prefer an invitation may be patients who do not take the initiative to ask questions about their concerns. It is beyond the scope of this study to determine if patients with a life-threatening illness like metastatic melanoma can take the initiative to raise concerns along the patient pathway. Likewise, to determine whether all items are equally important to all kinds of patient groups. However, this could be an important point to investigate in future research.

As several patients in the intervention group did not feel that physicians invited them to ask questions and discuss their concerns, this raises doubts about whether the intervention was delivered as intended. A small intervention fidelity study was conducted, demonstrating that the intervention was delivered as planned [32]. The physicians in the intervention group received a 1-h training session, ad hoc training, and a manual that was available during consultations [21]. Whether this approach adequately prepared the physicians for the intervention requires further investigation among the participating physicians, which was not the focus of this study.

In addition to preparing physicians to use the dialog tool, patients are also a central factor in its use, particularly for those who may perceive the intervention as a new way of interacting with their physicians. In the current study, patients are not systematically trained to use PRO actively in the consultation. This could be of future interest. Furthermore, PRO may not be suitable for all patients; completion of questionnaires could be challenging for the elderly, cognitively impaired, very sick, and distressed patients, or patients with low literacy [11, 20]. However, this does not appear to be supported by the current study.

The active use of PROs during consultations can enhance patient–clinician communication by prompting discussion and streamlining consultations [11, 14, 15]. PRO tools may also provide physicians with a comprehensive understanding of the patient’s health status and offer valuable insights into the impact of the disease and treatment on the individual [13, 33]. Furthermore, the use of PRO in clinical practice may encourage patients to become more active participants in their healthcare, increasing their involvement due to greater awareness and reflection on symptoms and health, as well as reducing uncertainty about the importance of disclosing symptoms to their physician [11, 14, 20, 33]. The impact of the dialog tool on patients in the current study has not been explored but warrants further investigation. Additionally, exploring patients’ experiences with its use would be valuable for considering potential improvements.

It can be argued that the intervention investigated in this study aims for a higher degree of patient involvement than a generic measurement tool alone can provide, as patients are also asked to list their concerns and the most important topics to discuss during consultation. As described earlier, great demands are placed on patients with melanoma about managing their health, and the patients must be involved in decisions about their treatment. It could be decisions about pausing treatment due to experienced side effects or adjusting the treatment schedule to accommodate an important event the patient wishes to attend. Even though patients are provided with the opportunity to put this on the agenda via PRO, it does not lead to more involvement in decision-making (item 4) or communication regarding the management of illness (item 5), as the study finds no differences between the intervention- and control group in items 4 and 5.

### Strengths and limitations

A strength of the study is the use of a validated questionnaire to measure patient involvement [22]. The patients who chose to participate were homogeneous with regard to the collected baseline data, regardless of whether they were in the intervention or control group. They were likely highly motivated, as very few failed to complete the questionnaires during the project period. Their strong positivity toward patient involvement may explain the ceiling effect observed in the data, suggesting that our results may not represent the entire melanoma population [34]. Prominent barriers on an individual level to using PRO are lack of time, incapacity, and difficulty using electronic devices to complete PRO [35]. Therefore, some patients may not be suitable for a study like this. All data in this study is patient reported. This is relevant when it comes to patient involvement as PRO provide direct insight into how patients feel and function, which clinical or laboratory measures cannot fully assess. However, consequently, we do not have data on disease stage, performance status, nor subtype of treatment, which is of major interest clinically. These parameters could perhaps have an impact on the assessment of patient involvement. A limitation of our results in Figure 2 is that here 15 tests are performed, although they are likely not independent. Thus, the highlighted results may be random findings and furthermore biased by the difference in the number of participants at risk at different time-points since there is no adjustment in the tests. There may be room for debate regarding our choice to test. Perhaps it would be more appropriate to focus on trends in the figures rather than conducting statistical tests. Another limitation is that our results were based on a non-randomization design, which entails the risk of selection bias. Differences in treatment, care, health-seeking behavior, and attitude toward the patient–physician relationship may be present according to the different study sites, which may represent the risk of bias. It is known that despite Denmark being a small country, differences in mortality exist between the regions of Denmark [36]. Whether clinical parameters such as disease stage and performance status impact patients’ self-assessed involvement is unknown. The difference in time from diagnosis to inclusion between the intervention and control group may also represent challenges when interpreting the results. A strong patient–physician relationship could have been established before inclusion into the study, distorting the outcome. However, no development in patient involvement is detected during the project period indicating that a prior or longer relationship did not impact patient involvement in this study. Furthermore, the limited availability of secondary data hindered our ability to collect additional information, such as the number of consultations per patient, which could have been a confounder and strengthened our results.

## Study interpretation

The use of PRO as a dialog tool in consultations for patients with metastatic melanoma has positively impacted patient involvement. These findings highlight the potential of PRO to enhance patient activation and engagement in their care. However, further research is needed to explore the impact of increased patient involvement on health outcomes and its broader implications for clinical practice.

## Supplementary Material



## Data Availability

Data is available on request to the corresponding author.
